# The indispensable role of Mediator complex subunit 27 during neurodevelopment

**DOI:** 10.1186/s13578-025-01425-7

**Published:** 2025-06-16

**Authors:** Xiaocheng Li, Nuermila Yiliyaer, Tianyu Guo, Hui Zhao, Yong Lei, Shen Gu

**Affiliations:** 1 School of Biomedical Sciences, Faculty of Medicine, The Chinese University of Hong Kong (CUHK), Hong Kong SAR, China; 2 Gerald Choa Neuroscience Institute, CUHK, Hong Kong SAR, China; 3 CUHK Shenzhen Research Institute, Shenzhen, China; 4 Key Laboratory for Regenerative Medicine, School of Biomedical Sciences, Faculty of Medicine, Ministry of Education, CUHK, Hong Kong SAR, China; 5 CUHK-GIBH CAS Joint Research Laboratory on Stem Cell and Regenerative Medicine, CUHK, Hong Kong SAR, China; 6 Joint Laboratory of Bioresources and Molecular Research of Common Diseases, Kunming Institute of Zoology - The Chinese University of Hong Kong (KIZ-CUHK), Hong Kong SAR, China; 7 School of Medicine, The Chinese University of Hong Kong (Shenzhen), Shenzhen, Guangdong 518172 China; 8 Hong Kong Branch of CAS Center for Excellence in Animal Evolution and Genetics, The Chinese University of Hong Kong, Hong Kong SAR, China

**Keywords:** Mediator complex, MED27, Neurodevelopmental disorder, Zebrafish model

## Abstract

**Background:**

MED27 is a subunit of the Mediator complex, a highly conserved protein assembly that initiates transcription by bridging transcription factors bound at enhancers to RNA polymerase II transcription machinery at promoters. Recently, we identified an autosomal recessive neurodevelopmental disorder (NDD) caused by loss-of-function (LoF) variants in the *MED27* gene. Affected individuals exhibit global developmental delay, intellectual disability, dystonia, and cerebellar atrophy, highlighting the neuronal system’s vulnerability to *MED27* disruptions.

**Results:**

To investigate the pathogenicity mechanisms and essential roles of this gene during neurodevelopment, we generated multiple zebrafish lines with LoF mutations in *med27*. Homozygous mutant zebrafish displayed severe developmental defects, motor deficits, and cerebellar atrophy, recapitulating the clinical phenotypes observed in *MED27*-NDD patients. Rescue experiments revealed that patient-specific mutant *MED27* mRNA failed to restore normal phenotypes in mutant zebrafish, unlike wildtype *MED27* mRNA, underscoring the clinical relevance of our models. Molecular analysis identified transcription factors *foxo3a* and *fosab* as direct downstream targets of *med27*. These genes are well-established master regulators in the central nervous system, providing mechanistic insights into how *med27* disruption impairs neuronal and cerebellar development.

**Conclusion:**

Our findings establish *med27* as a critical gene of embryogenesis and neurogenesis, shedding light on the disease mechanism underlying *MED27*-associated NDDs.

**Supplementary Information:**

The online version contains supplementary material available at 10.1186/s13578-025-01425-7.

## Background

The Mediator complex (MED) is a highly conserved protein complex consisting of the head, middle, and tail modules. Twenty-six MED subunits have been identified in humans, including subunit MED27, which straddles the head and tail modules [1, 2]. MED serves as a transcription coactivator by bridging transcription factors (TFs) bound at enhancers to the RNA polymerase II (Pol II) transcription machinery at promoters, thereby initiating the transcription of most protein-coding genes and many non-coding RNAs [3, 4]. Additionally, MED associates with a four-subunit kinase module to fine-tune gene transcription [5]. Evidently, MED plays fundamental and extensive roles in regulating gene expression.

Interestingly, the function of MED exhibits cell type-specific patterns, as evidenced by its distinct promotor binding sites in different cells, resulting in cell type-specific modulation of gene expression [6, 7]. Furthermore, studies on individual MED subunits revealed that each interacts with different TFs, activating distinct downstream target gene sets [3]. Therefore, each subunit may influence specific cellular or developmental processes [8, 9].

Currently, there are few published studies on the MED27 subunit, and its functional characterization during development remains limited. Previous studies using CRISPR-mediated genome editing to knockout (KO) the gene led to embryonic lethality in *Drosophila* and chicken, although the phenotypic abnormalities in these animal models were not documented [10–12]. Another study employing chemically induced mutagenesis of *med27* in zebrafish showed that it affected the development of catecholaminergic neurons, with decreased amacrine cells and increased rod photoreceptor cells in the retina, while abnormalities in other organs remained elusive [13]. The mouse genome informatics (MGI) database documented that mice with homozygous *Med27* null alleles exhibited preweaning lethality with complete penetrance (MGI genotype ID: 6288571), although additional phenotypes are unavailable. Therefore, the function of Med27 during embryonic development and other developmental stages remains largely uncharacterized.

Recently, through clinical exome sequencing, we identified an autosomal recessive neurodevelopmental disorder (NDD) caused by pathogenic variants in the *MED27* gene [14]. Among our patient cohort, 16 similarly affected NDD individuals from 11 families were found to carry homozygous or compound heterozygous *MED27* variants. Of the 11 unique variants identified, three were frameshift (FS) and one involved a canonical splice site, indicating a loss-of-function (LoF) of MED27. These patients exhibited homogenous phenotypes, including intellectual disability, developmental delay, central hypotonia, distal limb spasticity/dystonic movement, delayed motor and speech development, and cerebellar hypoplasia observed via brain magnetic resonance imaging (MRI). Subsequently, a larger cohort study [15] documented 57 *MED27* patients from 30 families (including the 16 affected individuals from our initial report), who manifested a broad phenotypic continuum of NDD. Nevertheless, all *MED27* patients consistently exhibited cerebellar hypoplasia, with progressive cerebellar atrophy observed in those who underwent follow-up MRI scans. The predominant neuronal phenotype, particularly the cerebellar atrophy, highlighted the neuronal system and cerebellum’s vulnerability to MED27 dysfunction.

Based on the clinical presentations of *MED27* patients, we hypothesized that MED27 plays critical roles in controlling neuronal and cerebellar development. Inspired by the likely LoF effect of patient-specific variants, we utilized zebrafish with *med27* LoF to characterize its roles during embryogenesis and neuronal development. Zebrafish has been demonstrated to be an appropriate animal model for investigating NDDs [16], as orthologs of human neurological disease-associated genes, including MED27, are well conserved in zebrafish (Fig. S1A). Additional advantages include high-throughput analysis due to their large number of embryos and the ability to observe neuronal development in real-time due to their transparency. Using CRISPR/Cas9 genome editing, we generated three zebrafish lines with *med27* LoF and demonstrated the neuropathological, behavioral, and molecular abnormalities in these models. Our study not only underscored the essential roles of Med27 and identified its critical downstream targets during embryogenesis and cerebellar development, but also demonstrated that patient-specific mutant *MED27* mRNA failed to rescue the phenotypic abnormalities to the same extent as wildtype (WT) *MED27* mRNA, highlighting the clinical relevance of our models.

## Methods

### Zebrafish maintenance

Zebrafish were housed under standard conditions of lighting (14 h light/dark cycle, lights on between 9 am and 11 pm), pH 7.5, conductivity of 500 µS, and temperature (26–28 °C). They were fed using the robotic Tritone feeding system. All animal experiments were approved by the Animal Experimentation Ethics Committee (AEEC) at The Chinese University of Hong Kong (CUHK), and all the experimental procedures were conducted in the accordance of AEEC guidelines at CUHK.

### Generation of zebrafish with LoF of *med27*

The CRISPR/Cas9 system was used to introduce LoF alleles in the *med27* gene in zebrafish. sgRNAs were designed using the CRISPRscan website (https://www.crisprscan.org/), and the sgRNA with the highest cutting efficiency was selected (Supplementary Table 2). sgRNA was synthesized following published protocol [17], purified using the RNeasy^®^ MinElute Cleanup Kit (QIAGEN #74204), and the resulting PCR product was used as a template for in vitro transcription using the MAXIscript™ T7 Transcription Kit (Thermo Fisher #AM1314). Alt-R™ S.p. HiFi Cas9 Nuclease V3 protein was purchased from Integrated DNA Technologies Pte. Ltd (#1081061). The sgRNA (200 ng/µL) and Cas9 protein (10 µM) were mixed at a 1:1 volume ratio prior to embryonic injection.

WT zebrafish were bred in a breeding tank overnight, and the separator was removed the following morning. Embryos were collected, and 2 nL of the sgRNA/Cas9 mixture was microinjected into the yolk at the one- to four-cell stage. Genotyping of the edited zebrafish was performed by tail clipping when they reached adulthood. Mosaic founders (G0) with the desired FS mutations were crossed with WT fish to generate the F1 mutant line with heterozygous FS mutations. F1 mutant fish were then self-crossed to produce F2 offspring, which included WT, heterozygous, and homozygous larvae. Genotyping primers are listed in Supplementary Table 2.

### Synthesis of *med27*, *olig2*, and *pvalb7 *probes

Total RNA was extracted from WT zebrafish, and coding sequences (CDS) of the target genes were PCR amplified by reverse transcription (primer sequences are listed in Supplementary Table 2). The amplified cDNAs were ligated into the pBluescript II plasmid and linearized using the restriction enzymes EcoRI (NEB #R3101) or NotI (NEB #R3189). Anti-sense and sense probes were generated through reverse transcription and diluted to a concentration of 1 ng/µL in hybridization buffer for WISH (whole-mount in situ hybridization).

### WISH

Zebrafish embryos were collected at specific time points and fixed in 4% paraformaldehyde (PFA) overnight. The fixed embryos were washed with PBST three times, with their chorions removed manually using forceps. Embryos older than 24hpf were treated with proteinase K (10 µg/mL, Invitrogen #100005393). Pre-hybridization was performed at 65℃ for at least 4 h. WISH probes were then added, and embryos were incubated at 4℃ overnight. Subsequently, the embryos were washed twice in a stepwise manner with 50% formamide in 2xSSCT, 2xSSCT, and 0.2xSSCT at 65℃. After washing, embryos were incubated with blocking solution for 4 h at room temperature and then incubated overnight with anti-digoxigenin antibody (1:2500, Sigma #11093274910) at 4℃. Post antibody incubation, embryos were washed with PBST three times and stained with the P substrate. The reaction was stopped using 4% PFA when positive signals were observed. Images were captured using a stereo microscope. Embryos were then collected, and genomic DNA (gDNA) was extracted by lysis in 20 µL alkaline lysis buffer (25 mM NaOH and 0.2 mM EDTA) at 95℃ for 30 min. The products were neutralized with an equal volume of neutralization buffer (40 mM Tris-HCl), and genotyping was performed.

To quantify WISH staining signal intensity, images were converted to 8-bit grayscale in ImageJ and inverted. The signal region was selected using the polygon tool, and the signal intensity values were measured. For each image, the mean intensity of the stained region was calculated, and background intensity (measured in a non-stained area) was subtracted to obtain the final mean pixel intensity.

### RNA isolation and quantitative reverse transcription PCR (RT-qPCR)

Zebrafish larvae were euthanized using tricaine (MS222) and divided into head and tail sections for total RNA extraction and genotyping, respectively. Groups of 20 heads were collected from *med27*^*+/+*^, *med27*^*+/−*^, and *med27*^*−/−*^ larvae at 7dpf for RNA isolation. Total RNA was extracted with TRIzol™ Reagent (ThermoFisher #15596018) and purified using the TriRNA Pure Kit (Geneaid #TRPD200). Reverse transcription was performed to obtain cDNA and qPCR was conducted using SYBR Green Master Mix (ThermoFisher #01321882). Primer sequences for RT-qPCR are listed in Supplementary Table 2.

### RNA-sequencing (RNA-seq) analysis

Paired-end Illumina raw reads were trimmed using Trimmomatic-0.39 with the adapter reference TruSeq3-PE.fa:2:30:10 and aligned to the zebrafish genome (GRCz11.104) using Hisat2 v2.2.1. Read counts were generated with featureCounts v2.0.4 and used for differential gene expression analysis with DESeq2 v1.38.3 in R. DEGs were identified with an adjusted *P*-value cutoff of 0.05 and an absolute log_2_ fold change threshold of 1. The volcano plot was created using ggplot2 v3.5.1 in R. GO analysis was performed using the Database for Annotation, Visualization and Integrated Discovery (DAVID) online tool (http://david.ncifcrf.gov/). Gene Set Enrichment Analysis (GSEA) was conducted with clusterProfiler v4.10.1 in R.

### Dual-luciferase report assay

Promoter regions of *neurod1*, *gbx1*, *foxo3a*, *fosab*, and *en2a* were amplified from zebrafish gDNA and subcloned into the pGL3-basic luciferase report vector. The CDS of *med27* was amplified from zebrafish cDNA and subcloned into the pCS2 expression vector. The constructed plasmids were microinjected into WT embryos at one- to four-cell stage, with the Renilla plasmid co-injected as an internal control. The pCS2 backbone vector was co-injected with luciferase reporter plasmids in the control group. After 48 h, luciferase activity was measured using the Dual Luciferase Reporter Assay Kit (Vazmye #DL101) and recorded by the Thermo Varioskan LUX Multi-mode Microplate Reader.

### Behavioral analysis

Zebrafish larvae were placed individually in 48-well plates at 3 hpf. At 6 dpf and 7 dpf, motor behavior was recorded under a stereo microscope. Behavioral data were analyzed using EthoVision XT software (Noldus Information Technology BV). Larvae were subsequently euthanized, and gDNA was extracted for individual genotyping as described above.

### Survival analysis

Zebrafish larvae were placed individually in 48-well plates at 3 hpf and observed twice daily for mortality. The date of death was recorded, and deceased larvae were stored at -20 °C for subsequent genotyping. The observation process was conducted without external disturbance [18].

### Rescue experiments with human *MED27* mRNA

The CDS of WT *MED27* was amplified from human cell cDNA (primer sequences in Supplementary Table 2). Patient-specific mutant *MED27* CDS variants were generated using the Q5 Site-Directed Mutagenesis Kit (NEB #E0052) based on the WT CDS. Each CDS was subcloned into the pCS2 vector, linearized with EcoRI, and transcribed into mRNAs. Synthesized mRNAs were microinjected into mutant embryos at the one- to four-cell stage, and larvae were analyzed for morphological differences at 3 dpf.

### Morphology analysis

The sagittal and dorsal views of the larvae were captured under the microscope, and the images were imported into ImageJ with consistent scales. The body length (from head to tail without the fin), eye distance (straight-line distance between the edges of the eyes), cardiac region (enclosed in the edge of the heart), and eye area (enclosed in the edge of the eyes) were respectively measured and recorded.

### Morpholino knockdown (KD) of *med27*

*med27* and control morpholinos were synthesized by Gene Tools. Embryos were divided into four groups: blank (water injection), control morpholino, *med27* morpholino, and rescue (200 pg WT human *MED27* mRNA  and 8 ng *med27* morpholino). Reagents were microinjected at the one- to four- cell stage, and development was monitored for survival and deformation.

### Statistical analysis

Investigators were blinded to group assignment during data collection and analysis. Experiments were repeated at least three times. Data were analyzed using ImageJ and GraphPad Prism v8. Chi-square tests were used for mRNA rescue and morpholino experiments. One-way ANOVA was applied for WISH, behavior assays, RT-qPCR, and morphological changes. Unpaired t-test was used for expression levels of selected TFs. Mann Whitney test was used for dual-luciferase reporter assay. Data were presented as means ± standard deviation (SD). Statistical significance was defined as follows: *P* < 0.05 (*), *P* < 0.01 (**), *P* < 0.001 (***), *P* < 0.0001 (****), and *P* > 0.05 (ns, not significant).

## Results

### Generation of multiple lines of *med27 *LoF zebrafish

The zebrafish *med27* ortholog is located on chromosome 8 (NM_200660.1, GRCz11/danRer11) and exhibits a highly conserved amino acid sequence compared to humans (0% gap, 93.57% similarity, 88.42% identity, Fig. S1A). WISH analysis revealed ubiquitous expression of *med27* in WT zebrafish embryos from 4 h post-fertilization (hpf) to 36 hpf, with prominent expression in the brain region at 48 hpf (Fig. S1B).

**Fig. 1:**
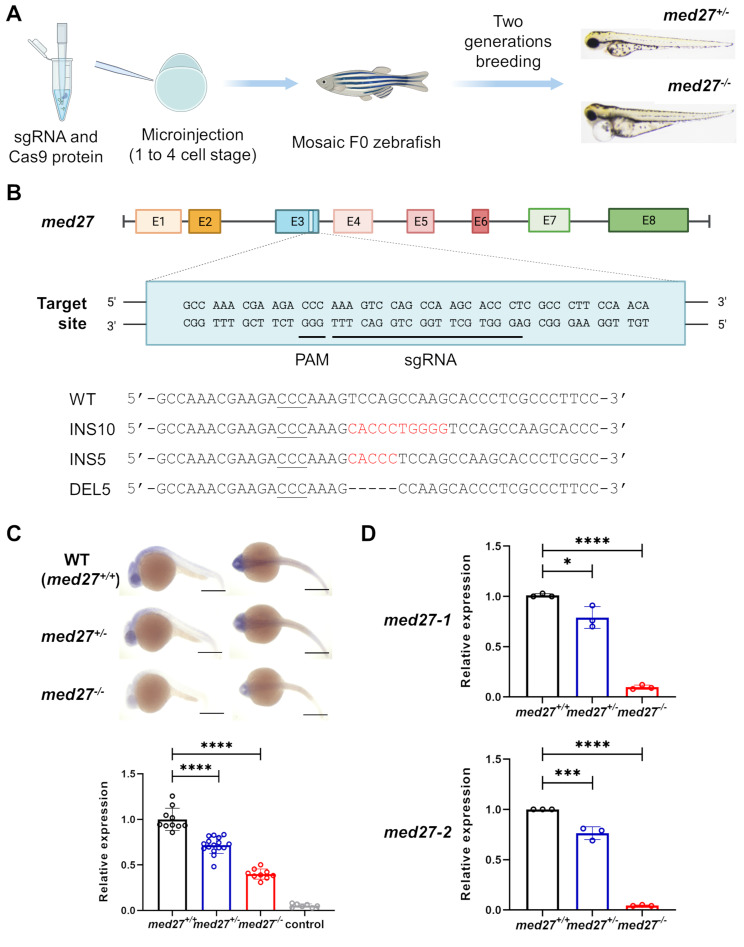
Generation of *med27 *LoF zebrafish. **A** Diagram illustrating the microinjection of the CRISPR/Cas9 genome editing system into zebrafish embryos to introduce *med27* null alleles. **B** Structure of the *med27* gene showing the sgRNA targeting site and the resulting *med27* frameshift mutations. E1 to E8: exons 1 to 8; PAM: protospacer adjacent motif; WT: wildtype; INS: insertion; DEL: deletion. **C** Representative lateral and dorsal views, along with quantification of *med27* expression in WT (*med27*^*+/+*^), *med27*^*+/−*^, and *med27*^*−/−*^ embryos, detected by WISH. Hybridization images were obtained using the *med27* antisense probe, with quantification shown for 24 hpf embryos from the INS10 line. For WISH results at various time points in all three lines, refer to Fig. S2A. Control: WISH with the sense probe. Scale bar = 500 μm. **D** Expression of *med27* in *med27*^*+/+*^, *med27*^*+/−*^, and *med27*^*−/−*^ larvae from the INS10 line at 7 dpf as detected by RT-qPCR. Two sets of primers (*med27*-1, located upstream of the induced mutation site, and *med27*-2, null downstream of the mutation site) were used in RT-qPCR to quantify *med27* mRNA. For RT-qPCR results in the INS5 and DEL5 lines, refer to Fig. S2B. Error bars represent mean ± standard deviation (SD). Statistical analysis was performed using one-way ANOVA. *: *P*<0.05; ***: *P*<0.001; ****: *P*<0.0001

To recapitulate the LoF variants identified in *MED27* patients and investigate the gene’s critical functions during development, we employed the CRISPR/Cas9 genome editing system [19] to generate *med27* knockout (KO) zebrafish (Fig. 1A). For effective gene KO, we strategically targeted the N-terminal-coding exons to induce LoF mutations while avoiding the first exon, as it may contain an additional initial codon downstream that maintains the open reading frame. We tested several single guide RNAs (sgRNAs) targeting exon 2 and 3 (data not shown) and ultimately selected the most efficient sgRNA targeting exon 3 to induce multiple lines with null *med27* alleles (Fig. 1B). Three lines with heterozygous FS mutations resulting in premature stop codons were chosen: a 10 base pairs (bp) insertion (INS10, c.448_449insCACCCTGGGG, p.V150Afs*17), a 5 bp insertion (INS5, c.448_449insCACCC, p.V150Afs*44), and a 5 bp deletion (DEL5, c.449_453delTCCAG, p.V150Afs*12). Sanger sequencing confirmed their heterozygous genotypes (Fig. 1B, S1C, collectively referred to as *med27*^*+/−*^).

To generate zebrafish with homozygous *med27* null alleles, *med27*^*+/−*^ fish were self-crossed to produce WT (*med27*^*+/+*^), heterozygous mutant (*med27*^*+/−*^), and homozygous mutant (*med27*^*−/−*^) offspring. WISH analysis was performed to examine *med27* expression in embryos of these different genotypes. Robust, ubiquitous expression was observed in 4 hpf and 6 hpf embryos irrespective of genotype, resembling the expression pattern in WT embryos (Fig. S1B, S2A). From 12 hpf to 36 hpf, slightly reduced expression was observed in *med27*^*+/−*^ embryos, while *med27*^*−/−*^ embryos exhibited significantly reduced expression (Fig. 1C, S2A). This reduction was further confirmed by RT-qPCR analysis in mutant larvae, indicating nonsense-mediated mRNA decay due to the FS mutations of *med27*. Two sets of primers (*med27*-1, located upstream of the induced insertion or deletion sites, and *med27*-2, downstream of the mutation sites) were used to quantify *med27* mRNA in 7 days post-fertilization (dpf) fish. Consistent with WISH results, *med27*^*+/−*^ fish showed a slight reduction in expression, whereas *med27*^*−/−*^ larvae exhibited markedly lower expression levels (Fig. 1D, S2B). These findings demonstrate the successful establishment of multiple zebrafish lines harboring *med27* LoF mutations.

### Med27 LoF zebrafish exhibited severe developmental defects, motor deficits, and shortened lifespan

**Fig. 2:**
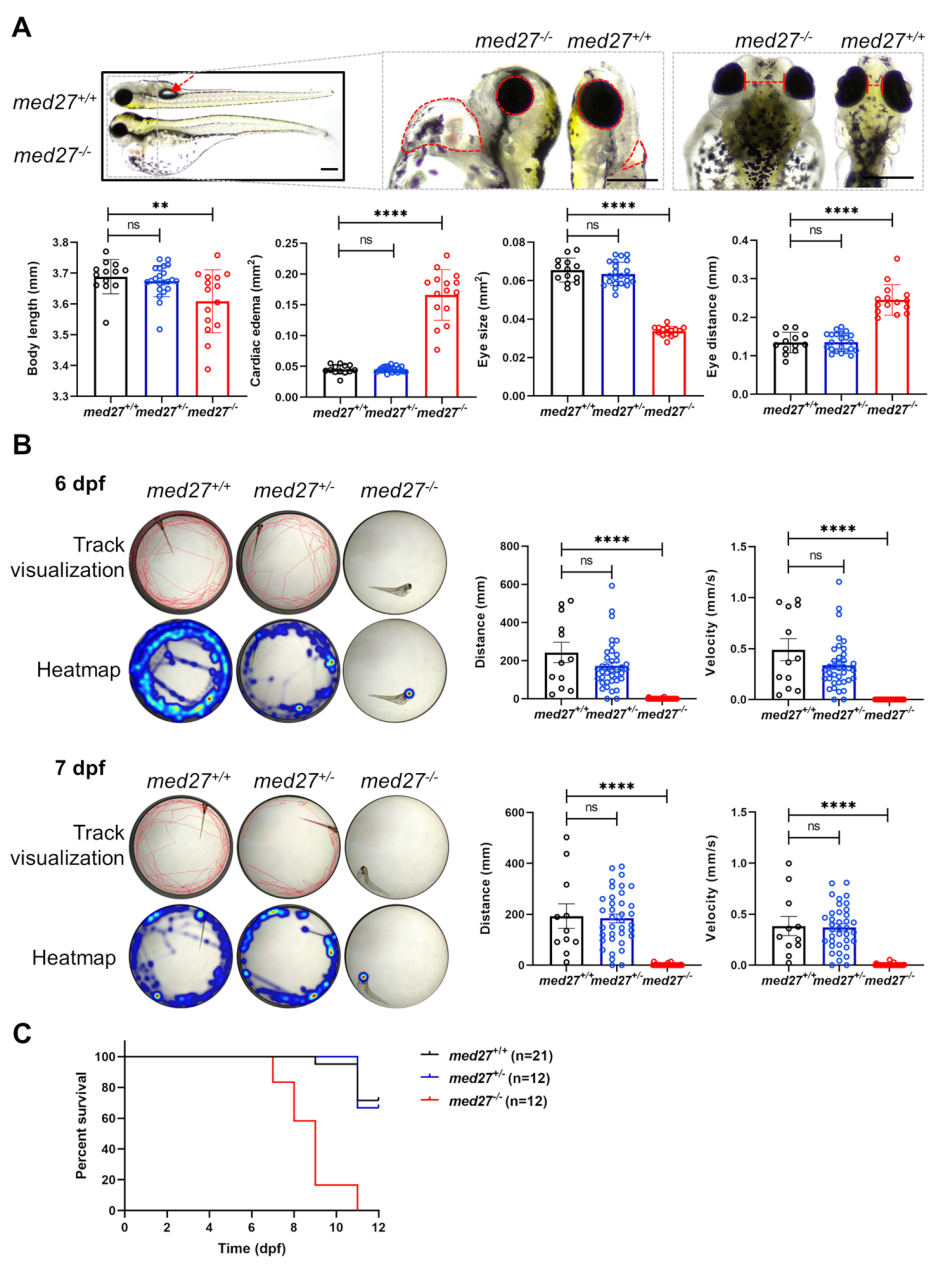
*med27 *LoF zebrafish exhibited severe developmental defects, motor deficits, and shortened lifespan. The results shown here are from the INS10 mutant line. For results from the INS5 and DEL5 mutant lines, refer to Fig. S3 and S4. **A** Representative images and quantitative comparisons of morphological changes in *med27*^*+/+*^, *med27*^*+/−*^, and *med27*^*−/−*^ larvae at 7 dpf (*med27*^*+/+*^, *n* = 13; *med27*^*+/−*^, *n* = 22; *med27*^*−/−*^, *n* = 15). Scale bar = 500 μm. Left panel: the dashed red arrow indicates the bladder; middle panel: the dashed circles highlight the heart and eye areas; right panel: the dashed line represents the eye distance. **B** Representative track visualizations and heatmaps showing swimming behavior of *med27*^*+/+*^, *med27*^*+/−*^, and *med27*^*−/−*^ larvae at 6 dpf (upper panel) and 7 dpf (lower panel). Corresponding quantifications of swim distance and velocity are provided (at 6 pdf, *med27*^*+/+*^*n* = 12, *med27*^*+/−*^*n* = 38, *med27*^*−/−*^*n* = 34; at 7 pdf, *med27*^*+/+*^*n* = 11, *med27*^*+/−*^*n* = 37, *med27*^*−/−*^*n* = 30). **C** Survival curve of *med27*^*+/+*^, *med27*^*+/−*^ and *med27*^*−/−*^ fish. Error bars represent mean ± SD. Statistical analysis was performed using one-way ANOVA. ns: not significant; **: *P*<0.01; ****: *P*<0.0001

From 2 dpf onward, distinct phenotypic abnormalities became evident in *med27*^*−/−*^ larvae compared to WT fish, and these differences progressively worsened. By 7 dpf, *med27*^*−/−*^ larvae displayed shortened body length, smaller eyes, increased eye distance, and cardiac edema compared to WT littermates (Fig. 2A, S3A). Homozygous mutant fish exhibited the absence of the swim bladder and curvature of the body axis, which were not observed in WT or heterozygous fish. Comparative analysis revealed significant phenotypic differences between homozygous mutants and WT fish, but not between heterozygotes and WT fish (Fig. 2A, S3A), consistent with the phenotypically normal parents of *MED27* patients carrying heterozygous LoF variants [14].

Motor behaviors were assessed by tracking swimming patterns. WT and *med27*^*+/−*^ larvae swam freely, whereas *med27*^*−/−*^ larvae struggled to move their tails and displayed minimal spontaneous movement (Fig. 2B). Swimming behavior was recorded and visualized through heatmaps, and swimming distance and velocity were quantitatively measured. The motor deficits observed exclusively in homozygous KO fish were consistent across all three mutant lines (Fig. 2B, S4).

Due to increased mortality in *med27*^*−/−*^ fish, survival rates were examined without feeding. Nearly all homozygous mutants died around 10 dpf due to severe deformities, such as pericardium edema and abdominal rupture, leading to significantly shorter survival periods compared to WT and *med27*^*+/−*^ larvae (Fig. 2C, S3B). At 9 dpf, the mortality rate among homozygous mutants reached 80%, whereas no WT and minimal *med27*^*+/−*^ larvae died by that point. All three mutant lines exhibited similar survival patterns, with *med27*^*−/−*^ fish showing earlier lethality compared to WT and *med27*^*+/−*^ fish. When comparing *med27*^*+/−*^ with WT fish, their survival rates were either indistinguishable (INS10 line, Fig. 2C) or slightly reduced (INS5 and DEL5 lines, Fig. S3B). Collectively, biallelic LoF of *med27* resulted in severely impaired embryonic development, morphological defects, loss of motor capacity, and reduced lifespan.

### Med27 LoF resulted in severe cerebellar atrophy

**Fig. 3:**
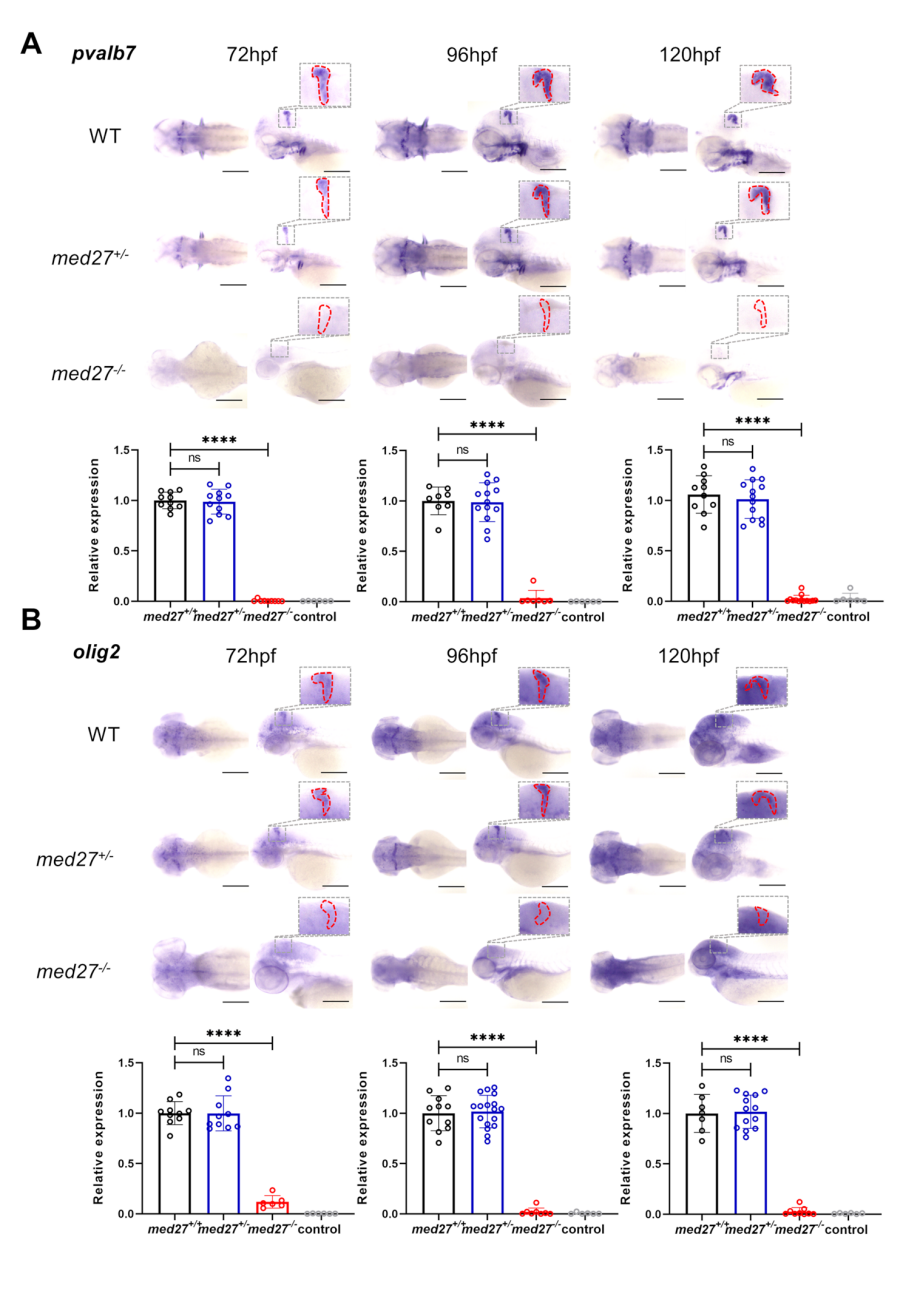
*med27 *LoF resulted in severe cerebellar atrophy. The results presented here are from the INS10 mutant line. For results from the INS5 and DEL5 mutant lines, refer to Fig. S5 and S6. Representative dorsal and lateral views of WISH for *pvalb7* (**A**) and *olig2* (**B**) at 72 hpf (*pavlb7*: *med27*^*+/+*^*n* = 10, *med2*^*+/−*^*n* = 11, *med27*^*−/−*^*n* = 9, control *n* = 6; *olig2*: *med27*^*+/+*^*n* = 10, *med27*^*+/−*^*n* = 10, *med27*^*−/−*^*n* = 6, control *n* = 6), 96 hpf (*pavlb7*: *med27*^*+/+*^*n* = 8, *med27*^*+/−*^*n* = 13, *med27*^*−/−*^*n* = 7, control *n* = 6; *olig2*: *med27*^*+/+*^*n* = 11, *med27*^*+/−*^*n* = 17, *med27*^*−/−*^*n* = 7, control *n* = 6), and 120 hpf (*pavlb7*: *med27*^*+/+*^*n* = 10, *med27*^*+/−*^*n* = 13, *med27*^*−/−*^*n* = 13, control *n* = 6; *olig2*: *med27*^*+/+*^*n* = 7, *med27*^*+/−*^*n* = 13, *med27*^*−/−*^*n* = 9, control *n* = 6) in larvae of the three genotypes. Enlarged views highlight the cerebellar region on lateral views enclosed by dashed red lines, which were quantified for gene expression comparison. Error bars represent mean ± SD. Statistical analysis was performed using one-way ANOVA. ns: not significant; ****: *P*<0.0001

Structural analysis of brain MRI revealed that all *MED27* patients consistently exhibited cerebellar atrophy, which is the most prominent clinical manifestation identified in these individuals [14, 15]. Consequently, we investigated the potential occurrence of cerebellar hypoplasia in the mutant zebrafish. We examined the expression levels of two cerebellar-specific markers, parvalbumin7 (*pvalb7*) and oligodendrocyte lineage transcription factor 2 (*olig2*), at different developmental stages using WISH (Fig. 3, S5, S6). *pvalb7* specifically labels inhibitory neurons known as Purkinje cells, the principal neurons of the cerebellum, while *olig2* labels excitatory neurons called eurydendroid cells, which provide cerebellar output [20, 21]. Since zebrafish cerebellum development begins at 2 dpf and achieves full circuitry and functionality by 6 dpf [22], we quantified the levels of *pvalb7* and *olig2* at 3, 4, and 5 dpf, respectively.

In all three mutant lines, both WT and *med27*^*+/−*^ fish exhibited robust signals for *pvalb7* and *olig2*, indicating normal gene expression. However, *med27*^*−/−*^ larvae displayed a complete loss of *pvalb7* and *olig2* expression at all examined time points, demonstrating severe cerebellar atrophy in the homozygous mutants. Expression levels of these two genes were further validated by RT-qPCR, which yielded results consistent with WISH (Fig. S5C, S6C). These findings illustrate the critical functional role of Med27 in cerebellum development.

### Deformities of *med27*^−/−^ fish rescued by WT human MED27 but not patient-specific mutant MED27

**Fig. 4:**
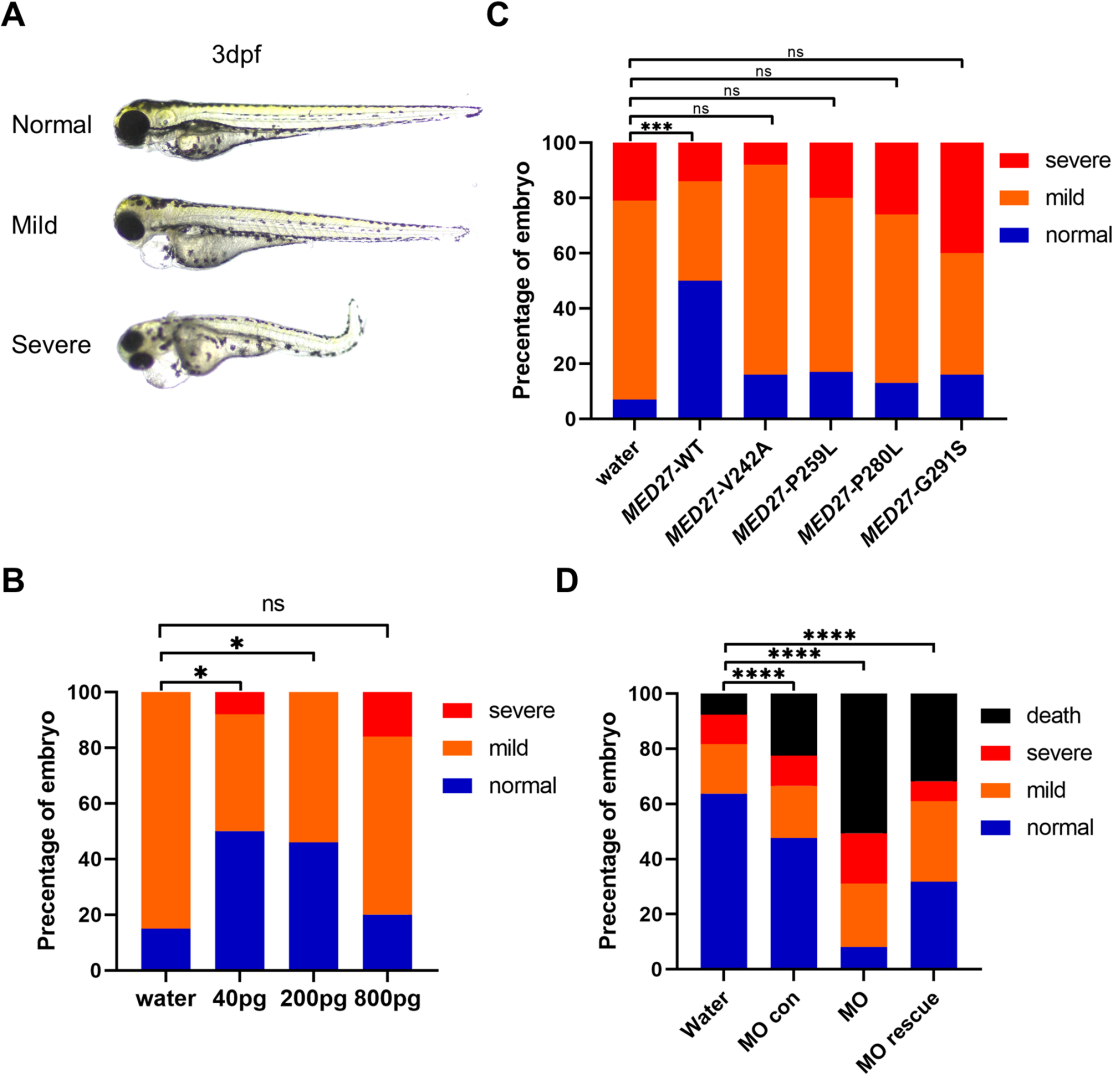
Rescue of *med27 *KO fish phenotype by human MED27. The results presented here are from the INS10 mutant line. For results from the INS5 and DEL5 mutant lines, refer to Fig. S7. **A** Three categories of phenotypes observed in larvae at 3 dpf after embryonic injection. **B** Distribution of larvae phenotypes after microinjection of different doses of WT *MED27* mRNA into *med27*^*−/−*^ embryos. The control group was injected with water (*n* = 20), and the following groups were injected with 40 pg (*n* = 24), 200 pg (*n* = 24), and 800 pg (*n* = 25) of WT *MED27* mRNA. **C** Distribution of phenotypes after rescue experiments using WT *MED27* mRNA or patient-specific mutant *MED27* mRNA. The water-injected group (*n* = 29) served as the control, while the following groups were injected with: *MED27*-WT (*n* = 28), *MED27*-V242A (*n* = 25), *MED27*-P259L (*n* = 24), *MED27*-P280L (*n* = 23), and *MED27*-G291S (*n* = 25). **D** Distribution of larval phenotypes after microinjection of water (*n* = 262), *med27* KD morpholino (MO group, *n* = 311), morpholino control (MO con group, *n* = 312), and *med27* KD morpholino plus WT *MED27* mRNA (MO rescue group, *n* = 283). Phenotype comparisons were analyzed using chi-square tests. ns: not significant; *: *P* < 0.05; ***: *P* < 0.001; ****: *P* < 0.0001

To investigate the functional effects of *MED27* variants identified in patients, we utilized homozygous mutant fish. Initially, we tested the phenotypic rescue capability of WT human MED27 by injecting WT *MED27* mRNA into embryos at the one- to four-cell stage. At 3 dpf, the injected embryos exhibited three categories of phenotypes: normal, mildly abnormal (with mild pericardial edema), and severely abnormal (with prominent pericardial edema and curvature of the body axis) (Fig. 4A). Different doses of WT *MED27* mRNA were tested, and the most effective dosage was determined to be 200 pg per embryo. At this dosage, nearly half of the injected fish exhibited a normal phenotype, while the remaining fish presented mild abnormalities, with no severely abnormal cases observed (Fig. 4B). Consequently, we used the 200 pg dosage for subsequent rescue experiments. Notably, although WT *MED27* mRNA partially rescued the phenotype of *med27*^*−/−*^ larvae, the larvae eventually succumbed to severe symptoms, indicating that the treatment only delayed the onset of the severe phenotype.

For functional testing, we selected the four most recurrent missense *MED27* variants: p.Gly291Ser (identified in 25 patients), p.Val242Ala (7 patients), p.Pro280Leu (6 patients), and p.Pro259Leu (3 patients) [15]. All four variants were found as homozygous changes in affected individuals. Injection of *MED27* mRNA containing these missense changes failed to rescue the zebrafish phenotype to the same degree as WT *MED27* mRNA, indicating functional deficits in these mutant MED27 proteins (Fig. 4C, S7).

Antisense morpholino (MO) oligonucleotides are commonly used for gene KD in zebrafish. By blocking translation and reducing endogenous protein expression, MOs serve as a complementary method to validate the effects observed in CRISPR/Cas9 genome-edited zebrafish. In our study, we designed a 25 bp MO targeting the *med27* mRNA at the translation initiation site to inhibit its expression. As shown in Fig. 4D, WT fish embryos injected with *med27* MO exhibited more severe deformities, higher morbidity, and increased mortality rates compared to embryos injected with water, with nearly no normal fish observed post-injection. These deformities resembled the abnormalities observed in *med27*^*−/−*^ larvae. Meanwhile, injection of WT human *MED27* mRNA partially rescued these deformities.

### Med27 LoF resulted in dysregulation of the transcriptional landscape related to neurodevelopment

**Fig. 5:**
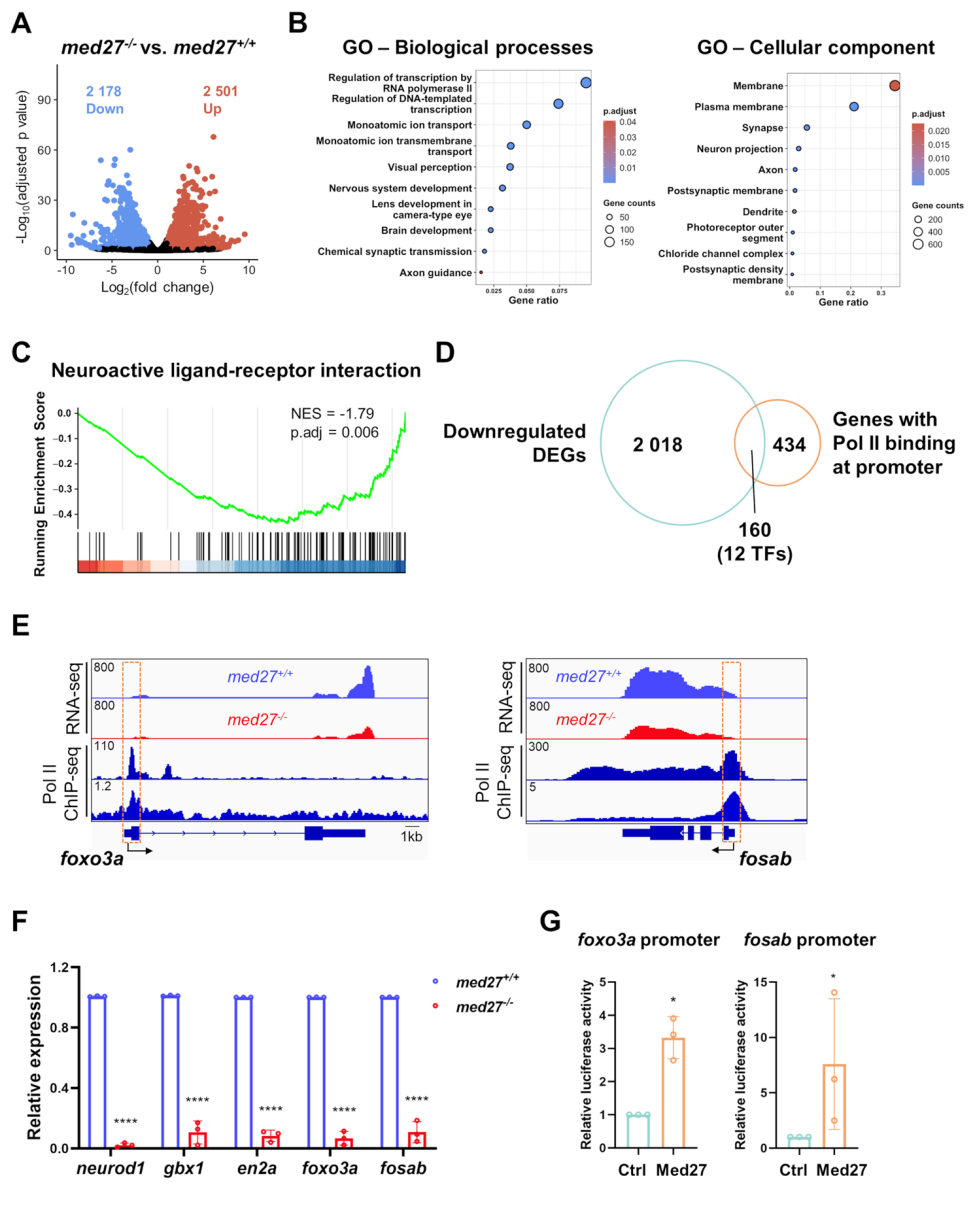
*med27* LoF resulted in dysregulation of the transcriptional landscape related to neurodevelopment. **A** Volcano plot showing RNA-seq results comparing the transcriptomic profiles of *med27*^*+/+*^ and *med27*^−/−^ larvae. RNA-seq was performed on pooled total RNA extracted from 7 dpf larvae from all three mutant lines (INS10, INS5, DEL5). 2 178 downregulated DEGs and 2 501 upregulated DEGs were identified (|Log_2_foldchange|>1, adjusted *P* < 0.05). **B** Top biological processes and cellular components identified by GO analysis of downregulated DEGs. The bubble size corresponds to the number of genes involved in each term, and the color intensity indicates the adjusted *P* value. **C** Gene set enrichment analysis (GSEA) plot showing enrichment of the neuroactive ligand-receptor interaction pathways among downregulated DEGs. **D** Venn diagram showing overlap of downregulated DEGs with positive Pol II binding at their promoter regions, based on Pol II ChIP-seq data (GEO Project ID PRJNA945049). TFs: transcription factors. **E** Bulk RNA-seq read counts of *foxo3a* (left) and *fosab* (right), along with the Pol II binding profiles at their promoter regions based on two Pol II ChIP-seq datasets (GEO Project ID PRJNA945049 and GSE175444). **F** RT-qPCR analysis of expression levels of identified overlapped TFs with well-established roles in brain and nervous system development. **G** Dual-luciferase reporter assays illustrating the activating effect of Med27 on promoter activities of *foxo3a* (left) and *fosab* (right). The assays were performed in zebrafish embryos. Ctrl: embryos injected with the pCS2 backbone vector; Med27: embryos injected with the pCS2-*med27* expression vector. Error bars represent mean ± SD. Statistical analysis was performed using unpaired t-test (Fig. 5F) or Mann Whitney test (Fig. 5G). *: *P* < 0.05; ****: *P*<0.0001

Given the essential role of MED in transcription initiation, we hypothesized that the neurodevelopmental abnormalities in Med27 LoF zebrafish were caused by transcriptional dysregulation of functionally critical genes. To investigate the underlying molecular mechanism, we performed transcriptomic profiling of WT and *med27*^*−/−*^ larvae at 7dpf using bulk RNA-seq, pooling total RNA extracted from all three mutant lines. We identified 2 178 downregulated differentially expressed genes (DEGs) and 2 501 upregulated DEGs (Fig. 5A). Gene ontology (GO) analysis of downregulated DEGs revealed enrichment of pathways involved in transcription regulation via Pol II, nervous system/brain/eye development, and synaptic transmission/axon guidance (Fig. 5B), consistent with the known function of MED and the abnormalities observed in mutant fish. Gene set enrichment analysis (GSEA) revealed an over-representation of downregulated DEGs enriched in neuroactive ligand-receptor interaction pathways, suggesting impairment of neuronal networks caused by *med27* disruption (Fig. 5C).

Since MED initiates transcription of downstream genes through Pol II within the transcription machinery at promoters, we analyzed publicly available ChIP-seq data for Pol II DNA binding profiles in developing zebrafish [23]. Among the 2 178 downregulated DEGs, 160 genes showed positive Pol II binding at their promoters, including 12 TFs (Fig. 5D, Supplementary Table 1). As TFs play pivotal roles in regulating gene expression, we specifically examined these dysregulated TFs. Among them, *neurod1*, *gbx1*, *foxo3a*, *fosab*, and *en2a* are critical for zebrafish neurodevelopment, as evidenced by their high expression in the central nervous system and their established roles in neurogenesis, brain patterning, and neuron survival [24–30]. To verify these findings, we conducted a similar analysis using another zebrafish Pol II ChIP-seq dataset (GEO #GSE175444) and consistently identified *gbx1*, *foxo3a*, and *fosab* as dysregulated TFs with positive Pol II binding at their promoters (Supplementary Table 1, Fig. 5E). The significantly reduced expression of these five TFs (*neurod1*, *gbx1*, *foxo3a*, *fosab*, and *en2a*) in *med27*^*−/−*^ larvae was confirmed by RT-qPCR (Fig. 5F). Dual-luciferase reporter assays performed in zebrafish embryos illustrated that induced expression of Med27 activated the promoter activity of *foxo3a* and *fosab*, validating the direct effect of Med27 on these two genes’ promoters (Fig. 5G). This activation effect was not observed for the promoters of *neurod1*, *gbx1*, or *en2a* (data not shown). Taken together, these findings support the interpretation that Med27 functions through *foxo3a* and *fosab* to regulate neurodevelopment in zebrafish, with Med27 LoF impairing the expression of these genes.

## Discussion

Besides *MED27*, several MED subunits genes, including *MED11* (Online Mendelian Inheritance in Man [OMIM] #620327), *MED17* (OMIM #613668), *MED23* (OMIM #614249), *MED25* (OMIM #616449), and the recently published *MED16* [31], have been associated with monogenic disorders. Additionally, subunits from the Mediator kinase module, including *MED12*, *MED12L*, *MED13*, *MED13L*, and *CDK8*, have been linked to Mendelian diseases (OMIM #301068/309520/300895/305450, 618872, 618009, 616789, and 618748, respectively). Collectively, these conditions are referred to as “neuro-MEDopathies”. Interestingly, all MED patients exhibit NDDs, albeit with differing severity, distinct neurological defects, and variable brain structural abnormalities. The consistent NDD phenotype underscores the essential roles of these MED subunits in regulating genes during neurodevelopment, while the variability in clinical presentations suggests that defects in individual MED subunits may dysregulate different downstream target genes and pathways.

Zebrafish have been widely used as an animal model to study functionally critical genes during neurodevelopment and to elucidate NDD pathogenicity, including *med11* and *med16* KO models [31, 32]. Several lines of evidence suggested a LoF disease mechanism for *MED27*-associated NDDs. First, the FS variants and variants disrupting canonical splicing sites identified in *MED27* patients are presumably LoF changes [14, 15]. In our current study, all three zebrafish lines carrying distinct *med27* FS mutations, which generate premature stop codons, exhibited an absence of *med27* expression due to nonsense-mediated mRNA decay, confirming LoF (Fig. 1, Fig. S2). Similar observations were made in human embryonic stem cells with FS *MED27* mutations, which we generated as preclinical cellular models of the disease using CRISPR-Cas9 genome editing (Yiliyaer, Li, & Guo et al., manuscript in revision). For the missense variants identified in *MED27* patients, our previous study demonstrated that patient-specific mutant MED27 resulted in decreased stability of the MED complex and reduced interactions between MED27 and its neighboring subunits, further supporting a LoF mechanism (Yiliyaer, Li, & Guo et al., manuscript in revision). Therefore, the LoF zebrafish models established in this study align with the proposed pathogenicity mechanism for *MED27*-NDD syndrome.

The three distinct *med27* LoF zebrafish lines we generated exhibited highly consistent abnormalities, cross-validating the robustness and reliability of our findings. Specifically, homozygous mutant fish from all three lines displayed shortened body length, reduced brain size, severe cerebellar atrophy, and minimal spontaneous swimming activity (Figs. 2 and 3 and S3-6). These anomalies are consistent with the clinical presentations of *MED27* patients, such as global developmental delay, short stature, structural brain abnormalities, progressive cerebellar atrophy, and motor deficits, all of which are associated with biallelic *MED27* pathogenic variants [14, 15]. In contrast, heterozygous mutant fish were comparable to WT fish in terms of morphology, cerebellar structures, and motor capacity, consistent with the observation that *MED27* patients’ parents or relatives, who are heterozygous carriers, being unaffected [14, 15].

In addition to the phenotypic similarities to patients, the clinical relevance of our transgenic zebrafish models was further demonstrated through rescue experiments using human MED27. Injecting an appropriate amount of WT *MED27* mRNA into mutant embryos resulted in half of the injected fish exhibiting normal phenotypes, while the remaining half showed mild anomalies; this contrasts with the nearly complete absence of normal fish in the control group injected with water or in test groups injected with patient-specific mutant *MED27* mRNAs (Fig. 4, Fig. S7). These data suggest that the mutant zebrafish models could be utilized to evaluate the pathogenicity of future *MED27* variants of uncertain clinical significance, providing functional evidence to assist in variant curation. This further highlights the clinical relevance of our models.

Although the embryonic lethality of Med27 KO by CRISPR genome editing in *Drosophila* and chicken was previously demonstrated, phenotypic abnormalities in these animal models were not analyzed [10–12]. A zebrafish study conducted two decades ago used random chemical mutagenesis to generate *med27* mutants [13], which is less precise than CRISPR-Cas9-induced *med27* mutations in our study. Moreover, the previous zebrafish study [13] focused only on retinal abnormalities, while potential defects in other organs were not investigated. Therefore, our study is the first to highlight the indispensable role of Med27 during embryogenesis and cerebellar development, which was largely uncharacterized prior to this analysis.

Our molecular investigation revealed how Med27 exhibits its essential role in embryonic neurogenesis and cerebellar development - by directly activating downstream master regulatory TFs critical for these processes (Fig. 5). We demonstrated that *med27* LoF causes a dysregulated transcriptomic landscape, with downregulated DEGs enriched in pathways related to Pol II-mediated transcription and neuronal system development. Among these DEGs, we pinpointed potential direct downstream master regulatory TFs of Med27 through integrative analysis with Pol II DNA binding profiles in developing zebrafish. Two candidate TFs, *foxo3a* and *fosab*, were validated as being directly regulated by Med27, providing evidence that Med27 functions through these genes to regulate neurodevelopment in zebrafish. Given the essential role of MED in initiating transcription of most protein-coding and non-coding genes, it is likely that Med27 disruption dysregulates multiple downstream target genes during key developmental processes.

Both *foxo3a* and *fosab* have well-established roles in neurodevelopment, as evidenced by studies in zebrafish, mice, and human cells. Specifically, *foxo3a* expression in zebrafish became confined to the central nervous system during embryonic development, and MO-mediated KD of the gene resulted in neurodevelopmental defects with increased neural apoptosis [29]. In mice, germline KO of *Foxo3* (the mouse ortholog of *foxo3a*) reduced the quiescence of neural stem cells (NSCs), leading to hyperproliferation and premature depletion of the NSC pool early in life. NSC-specific KO of *Foxo3* also induced lineage commitment bias toward astrocytes and away from oligodendrocytes and neurons [33]. In human cells, KD of another MED subunit, MED1, significantly reduced the expression of *FOXO3* (the human ortholog of *foxo3a*) [34], which is consistent with our findings.

*fosab*, the zebrafish orthologous of human *FOS*, belongs to immediate early gene (IEG) family, which plays critical roles in neuronal activity and brain development [35]. *fosab* is highly expressed in the zebrafish brain [36], and its disruption led to apoptosis and reduced cranial neural crest cells, resulting in craniofacial development abnormalities [37]. KO of *fosab* also caused deficits in learning and memory in zebrafish [27]. Interestingly, in cells from NDD patients with *MED23* or *MED12* pathogenic variants, *FOS* was identified as one of the top dysregulated genes [38, 39]. Furthermore, *FOS*, along with another IEG, *EGR1*, was identified as a direct downstream target of MED27 during early neurogenesis in our cellular models (Yiliyaer, Li, & Guo et al., manuscript in revision). These studies further strengthened our findings in zebrafish and suggested that *med27* LoF caused neurodevelopment defects by regulating the expression of *foxo3a* and *fosab*.

## Conclusions

In conclusion, we generated multiple zebrafish lines with Med27 LoF, all of which exhibited identical morphological and motor deficits consistent with the phenotypes of *MED27*-NDD patients. These models can be used to evaluate the pathogenicity of future *MED27* variants of uncertain clinical significance. Furthermore, our study highlights the essential functions of Med27 during embryonic and cerebellar development, potentially through the direct transcriptional activation of master regulatory TFs such as *foxo3a* and *fosab*. Our findings shed light on the disease mechanisms underlying *MED27*-associated NDDs and provide insights into investigating NDDs caused by pathogenic variants in other Mediator subunits.

## Electronic supplementary material

Below is the link to the electronic supplementary material.

Supplementary Material 1

Supplementary Material 2

Supplementary Material 3

## Data Availability

The ChIP-seq data utilized in this study are publicly available (GEO Project ID PRJNA945049 and GSE175444). The bulk RNA-seq data generated in the current study is available from the corresponding authors upon reasonable request. All other data generated or analyzed during this study are included in this published article and its supplementary information files.

## Abbreviations

MED: Mediator complex
MED27: Mediator complex subunit 27
Pol II: RNA polymerase II
TFs: Transcription factors
LoF: Loss-of-function
KO: Knockout
KD: Knockdown
MO: Morpholino
FS: Frameshift
WT: Wildtype
sgRNA: Single guide RNA
NDD: Neurodevelopmental disorder
MRI: Magnetic resonance imaging
WISH: Whole-mount in situ hybridization
AEEC: Animal experimentation ethics committee
DAVID: Database for annotation, visualization and integrated discovery
GSEA: Gene set enrichment analysis
DEGs: Differentially expressed genes
GO: Gene ontology
PFA: Paraformaldehyde
CDS: Coding sequence
gDNA: Genomic DNA
RT-qPCR: Quantitative reverse transcription PCR
RNA-seq: RNA-sequencing
hpf: Hours post-fertilization
dpf: Days post-fertilization
pvalb7: Parvalbumin 7
olig2: Oligodendrocyte lineage transcription factor 2
IEGs: Immediate early genes
fosab: v-fos FBJ murine osteosarcoma viral oncogene homolog Ab
foxo3a: Forkhead box O3A
