# Supplementary material for: The indispensable role of Mediator complex subunit 27 during neurodevelopment
Source: Cell Biosci. 2025 Jun 16;15:83. doi: 10.1186/s13578-025-01425-7 (PMC12172316; doi:10.1186/s13578-025-01425-7)
Supplement: Supplementary file 3 — Supplementary Material 3 [file 13578_2025_1425_MOESM3_ESM.pdf]

The indispensable role of Mediator complex subunit 27 during neurodevelopment

Xiaocheng Li[1], Nuermila Yiliyaer[1], Tianyu Guo[1], Hui Zhao[1,4,5,6,8], Yong Lei[7,*], Shen Gu[1,2,3,4,5,6,*]

[1]School of Biomedical Sciences, Faculty of Medicine, The Chinese University of Hong Kong (CUHK), Hong Kong SAR, China

[2]Gerald Choa Neuroscience Institute, CUHK, Hong Kong SAR, China

[3]CUHK Shenzhen Research Institute, Shenzhen, China

[4]Key Laboratory for Regenerative Medicine, Ministry of Education, School of Biomedical Sciences, Faculty of Medicine, CUHK, Hong Kong SAR, China

[5]CUHK-GIBH CAS Joint Research Laboratory on Stem Cell and Regenerative Medicine, CUHK, Hong Kong SAR, China

[6]Kunming Institute of Zoology - The Chinese University of Hong Kong (KIZ-CUHK) Joint Laboratory of Bioresources and Molecular Research of Common Diseases, Hong Kong SAR, China

[7]School of Medicine, CUHK (Shenzhen), Shenzhen, Guangdong 518172, China

[8]Hong Kong Branch of CAS Center for Excellence in Animal Evolution and Genetics, The Chinese University of Hong Kong, Hong Kong SAR, China

*Correspondence:

Shen Gu, shengu@cuhk.edu.hk

Yong Lei, leiyong@cuhk.edu.cn

SUPPLEMENTARY FIGURES

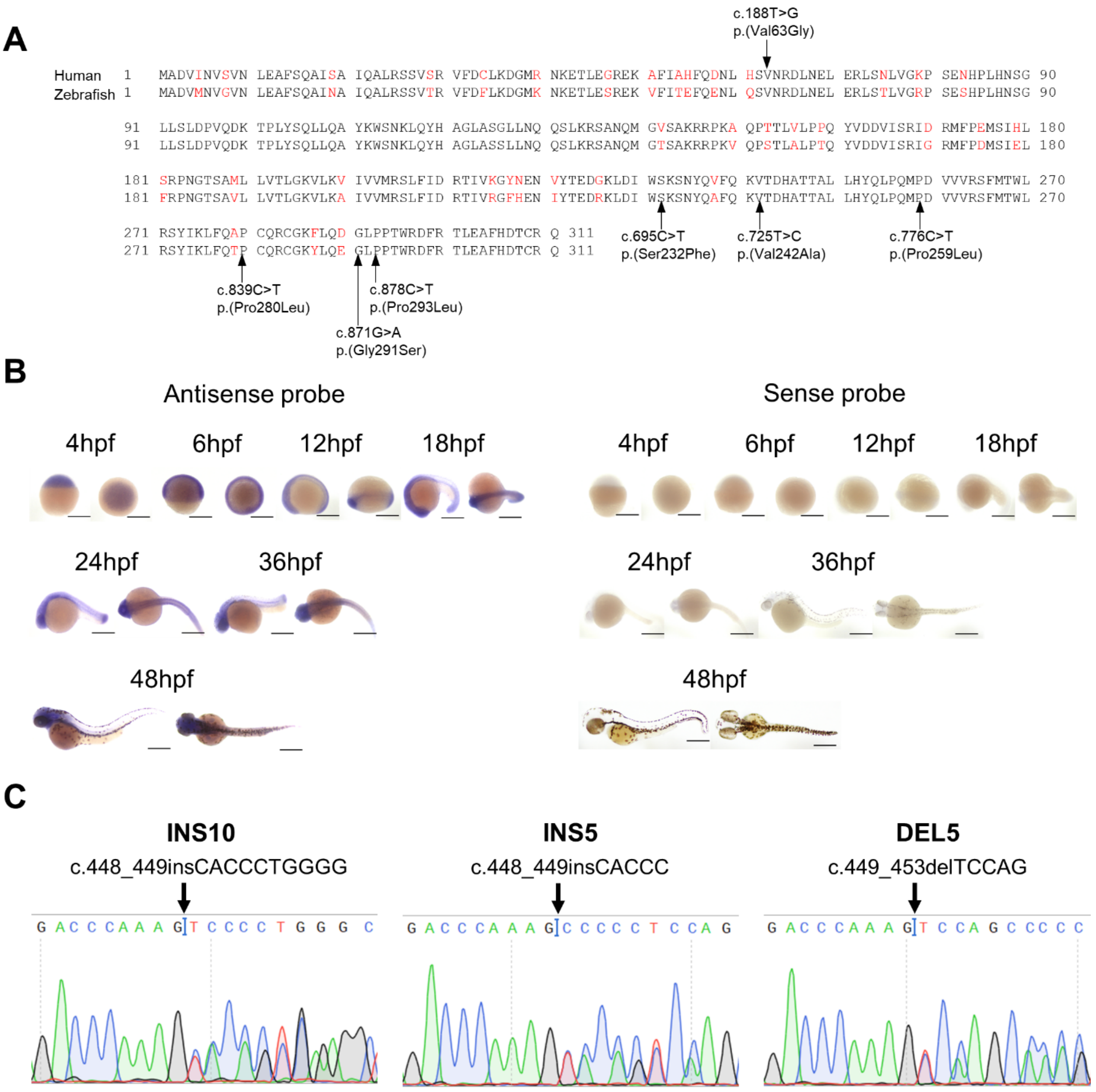

Fig. S1 Related to Fig. 1A a 1B. A Alignment comparison of human MED27 and zebrafish Med27 amino acid sequences. Differences in amino acids between the two species are highlighted in red. Black arrows indicate the missense variants identified in patients (Ref. 14). **B** Representative WISH images of WT zebrafish embryos hybridized with *med27* antisense or control sense probes. At least 36 embryos were analyzed, and all exhibited consistent expression patterns at each time point. Scale bar = 500 μm. **C** Sanger sequencing chromatograms confirming the null mutations generated in the three mutant lines. Arrows indicate the insertion or deletion sites.

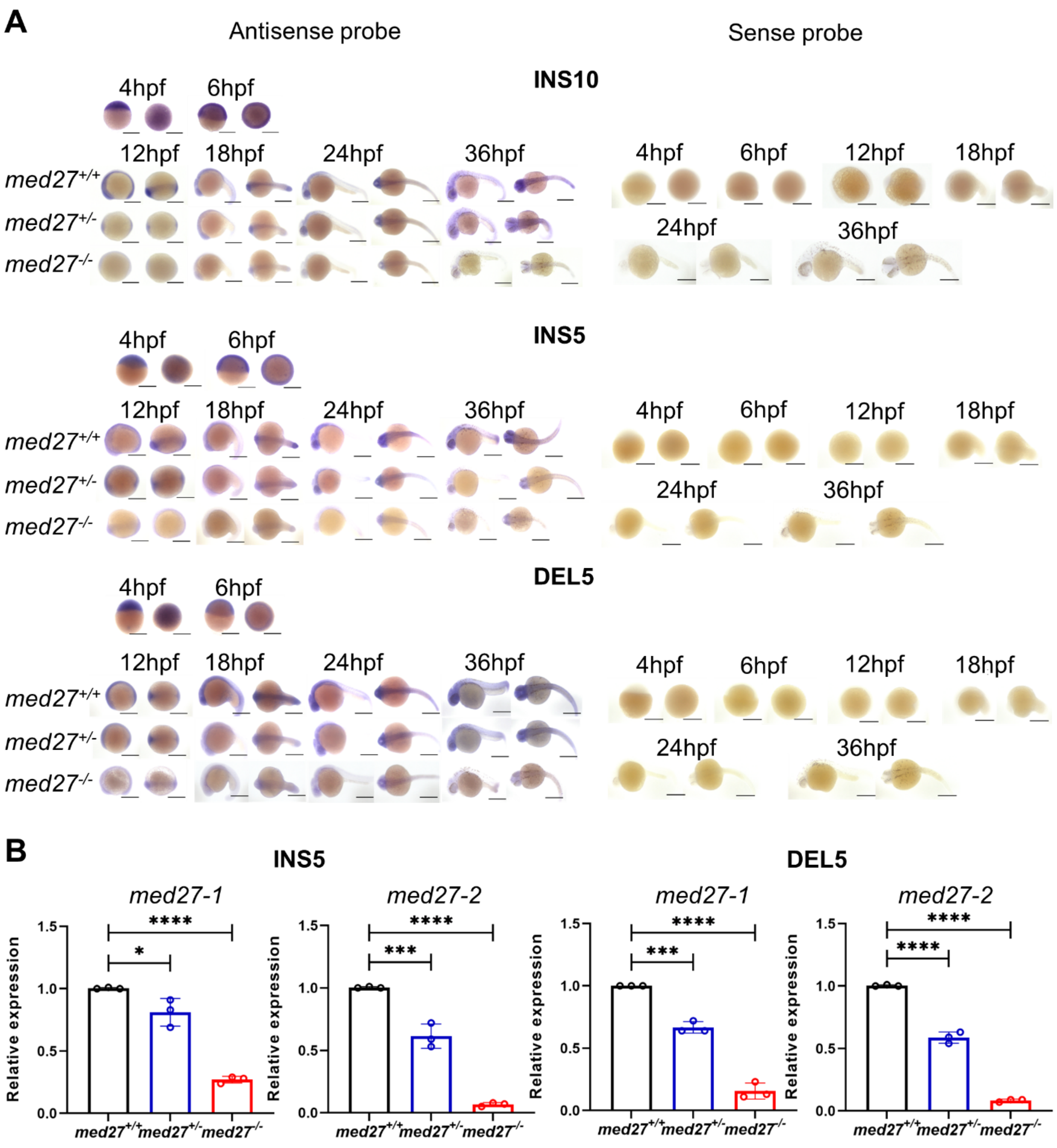

Fig. S2 Related to Fig. 1C and 1D. A WISH results showing the expression levels of *med27* in the three mutant lines across different developmental stages. Scale bar = 500 μm. **B** RT-qPCR analysis of *med27* expression in INS5 and DEL5 lines. Error bars indicate mean ± standard deviation (SD). Statistical analysis was performed using one-way ANOVA. *: $P < 0.05$; ***: $P < 0.001$; ****: $P < 0.0001$.

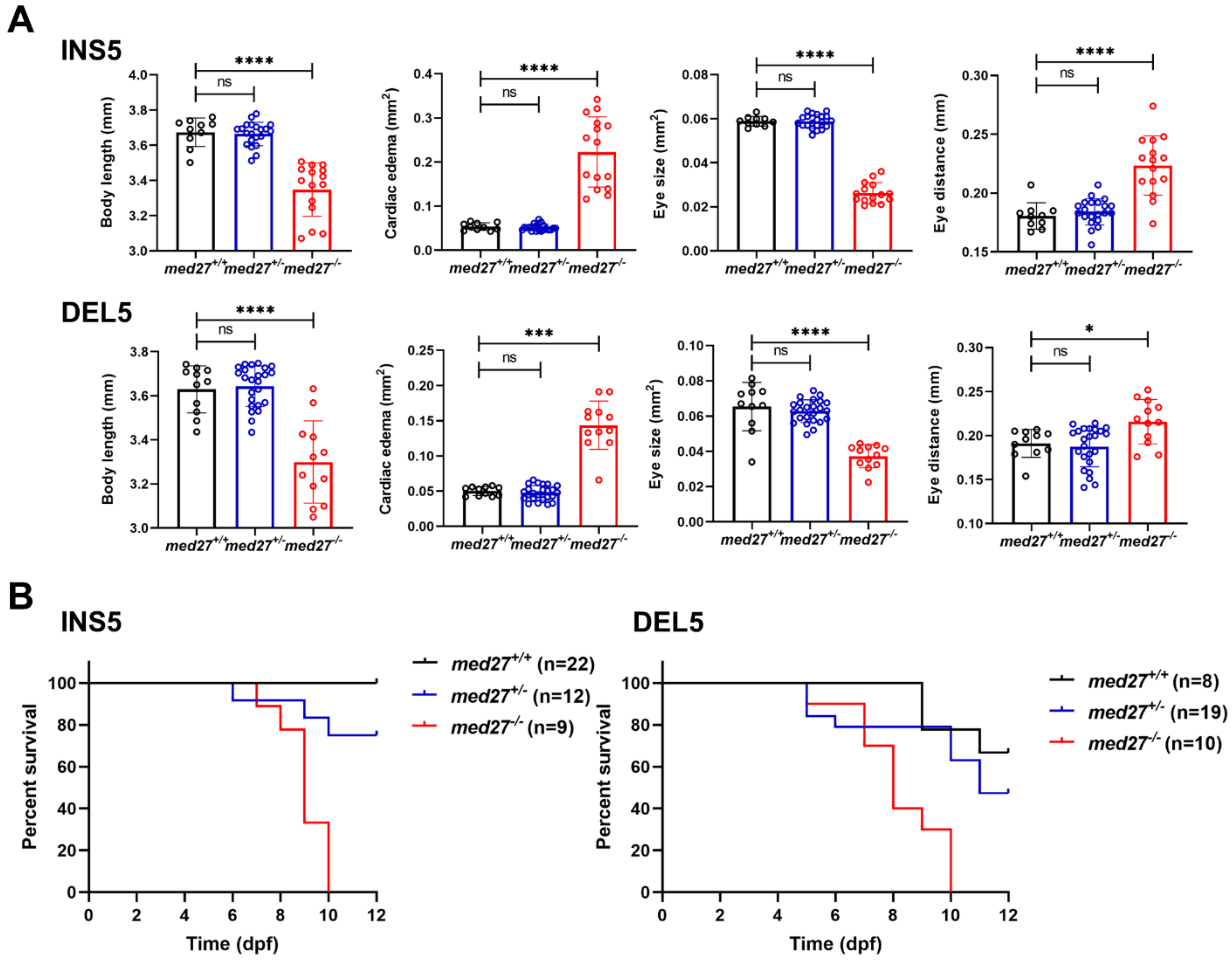

Fig. S3 Related to Fig. 2A and 2C. A Quantitative comparison of morphological changes in *med27*$^{+/+}$, *med27*$^{+/-}$, and *med27*$^{-/-}$ larvae at 7 dpf for the INS5 (*med27*$^{+/+}$ n=10, *med27*$^{+/-}$ n=21, *med27*$^{-/-}$ n=15) and DEL5 (*med27*$^{+/+}$ n=11, *med27*$^{+/-}$ n=24, *med27*$^{-/-}$ n=12) mutant lines. Error bars represent mean ± SD. Statistical analysis was performed using one-way ANOVA. ns: not significant; *: P<0.05; ***: P < 0.001; ****: P < 0.0001. **B** Survival curves of *med27*$^{+/+}$, *med27*$^{+/-}$ and *med27*$^{-/-}$ fish for the INS5 and DEL5 mutant lines.

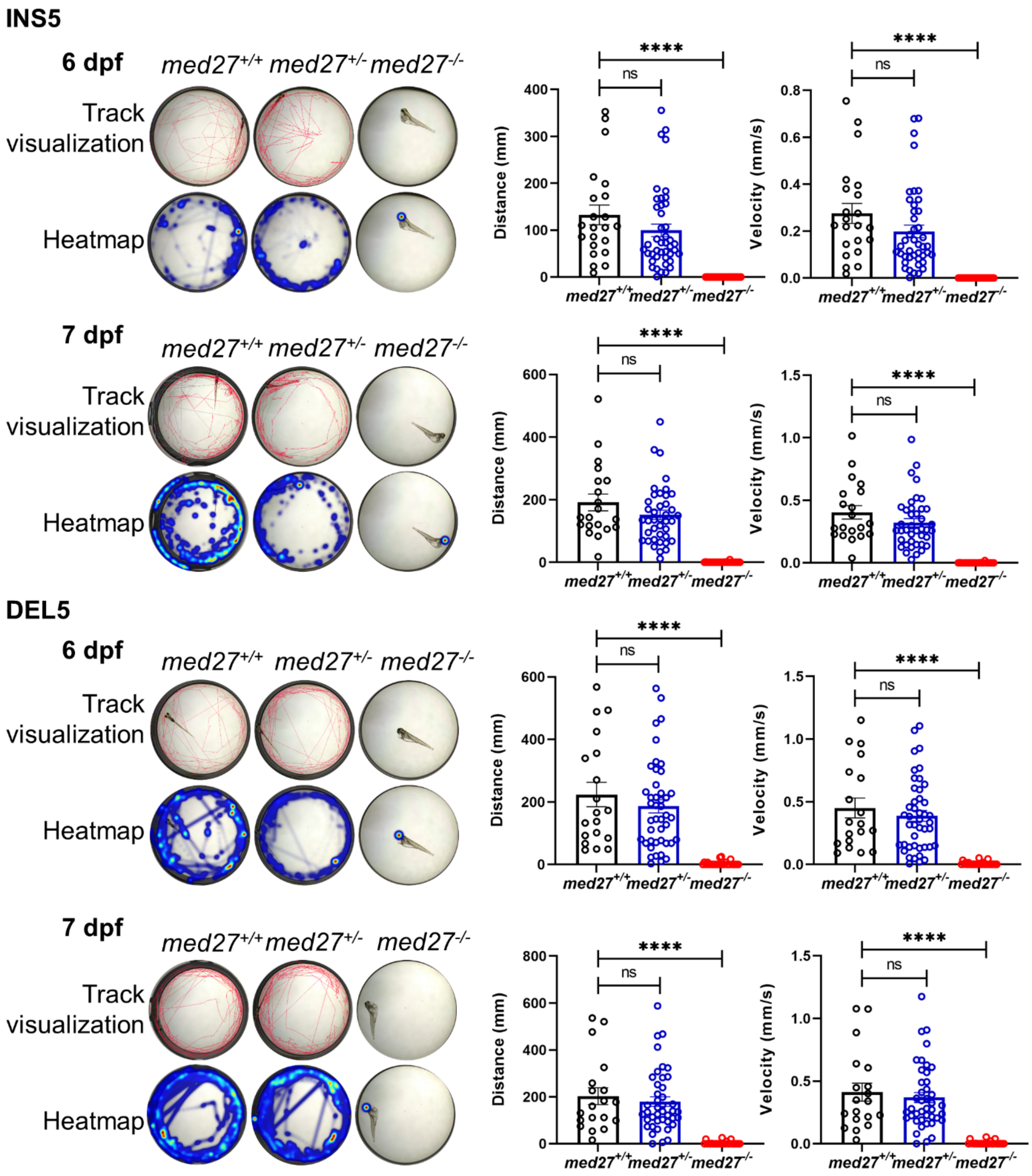

Fig. S4 Related to Fig. 2B. Representative track visualizations and heatmaps showing swimming behavior of *med27*$^{+/+}$, *med27*$^{+/-}$, and *med27*$^{-/-}$ larvae at 6 dpf and 7 dpf for the INS5 mutant line (upper panel) and the DEL5 mutant line (lower panel). Corresponding quantifications of swim distance and velocity were performed (INS5 at 6 dpf: *med27*$^{+/+}$ n=22,

med27$^{+/-}$ n=43, *med27*$^{-/-}$ n=25; INS5 at 7 dpf: *med27*$^{+/+}$ n=20, *med27*$^{+/-}$ n=40, *med27*$^{-/-}$ n=25; DEL5 at 6 dpf: *med27*$^{+/+}$ n=19, *med27*$^{+/-}$ n=44, *med27*$^{-/-}$ n=26; DEL5 at 7 dpf: *med27*$^{+/+}$ n=19, *med27*$^{+/-}$ n=42, *med27*$^{-/-}$ n=26). Error bars represent mean ± SD. Statistical analysis was performed using one-way ANOVA. ns: not significant; ****: $P < 0.0001$.

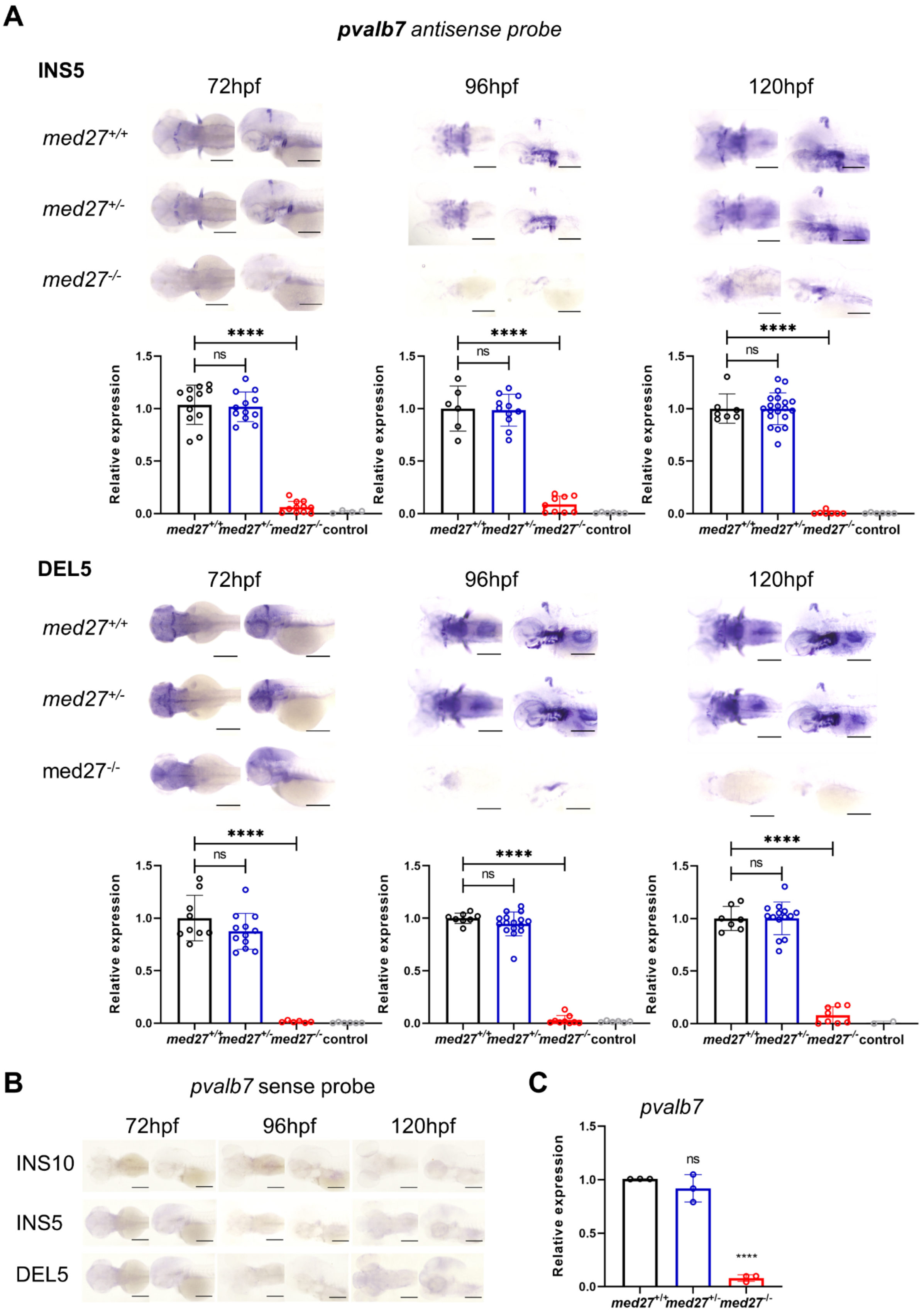
A
pvalb7 antisense probe
INS5
72hpf
96hpf
120hpf
med27+/+
med27+/-
med27-/-
****
ns
Relative expression
1.5
1.0
0.5
0.0
med27+/+ med27+/- med27-/- control
DEL5
B
pvalb7 sense probe
INS10
INS5
DEL5
C
pvalb7
med27+/+ med27+/- med27-/-

Fig. S5 Related to Fig. 3A. A Representative dorsal and lateral views of WISH for *pvalb7* at 72 hpf, 96 hpf, and 120 hpf in *med27*$^{+/+}$, *med27*$^{+/-}$, and *med27*$^{-/-}$ larvae from the INS5 and DEL5 mutant lines (INS5 at 72 hpf: *med27*$^{+/+}$ n=12, *med27*$^{+/-}$ n=12, *med27*$^{-/-}$ n=11, control n=6; INS5 at 96 hpf: *med27*$^{+/+}$ n=6, *med27*$^{+/-}$ n=11, *med27*$^{-/-}$ n=9, control n=6; INS5 at 120 hpf: *med27*$^{+/+}$ n=7, *med27*$^{+/-}$ n=20, *med27*$^{-/-}$ n=7, control n=6; DEL5 at 72 hpf: *med27*$^{+/+}$ n=9, *med27*$^{+/-}$ n=12, *med27*$^{-/-}$ n=6, control n=6; DEL5 at 96 hpf: *med27*$^{+/+}$ n=8, *med27*$^{+/-}$ n=16, *med27*$^{-/-}$ n=9, control n=6; DEL5 at 120 hpf: *med27*$^{+/+}$ n=7, *med27*$^{+/-}$ n=14, *med27*$^{-/-}$ n=8, control n=6). **B** WISH images with the *pvalb7* sense probe as controls. **C** mRNA expression levels of *pvalb7* in pooled total RNA extracted from 7 dpf larvae from all three mutant lines (INS10, INS5, DEL5). Error bars represent mean ± SD. Statistical analysis was performed using one-way ANOVA. ns: not significant; ****: $P < 0.0001$.

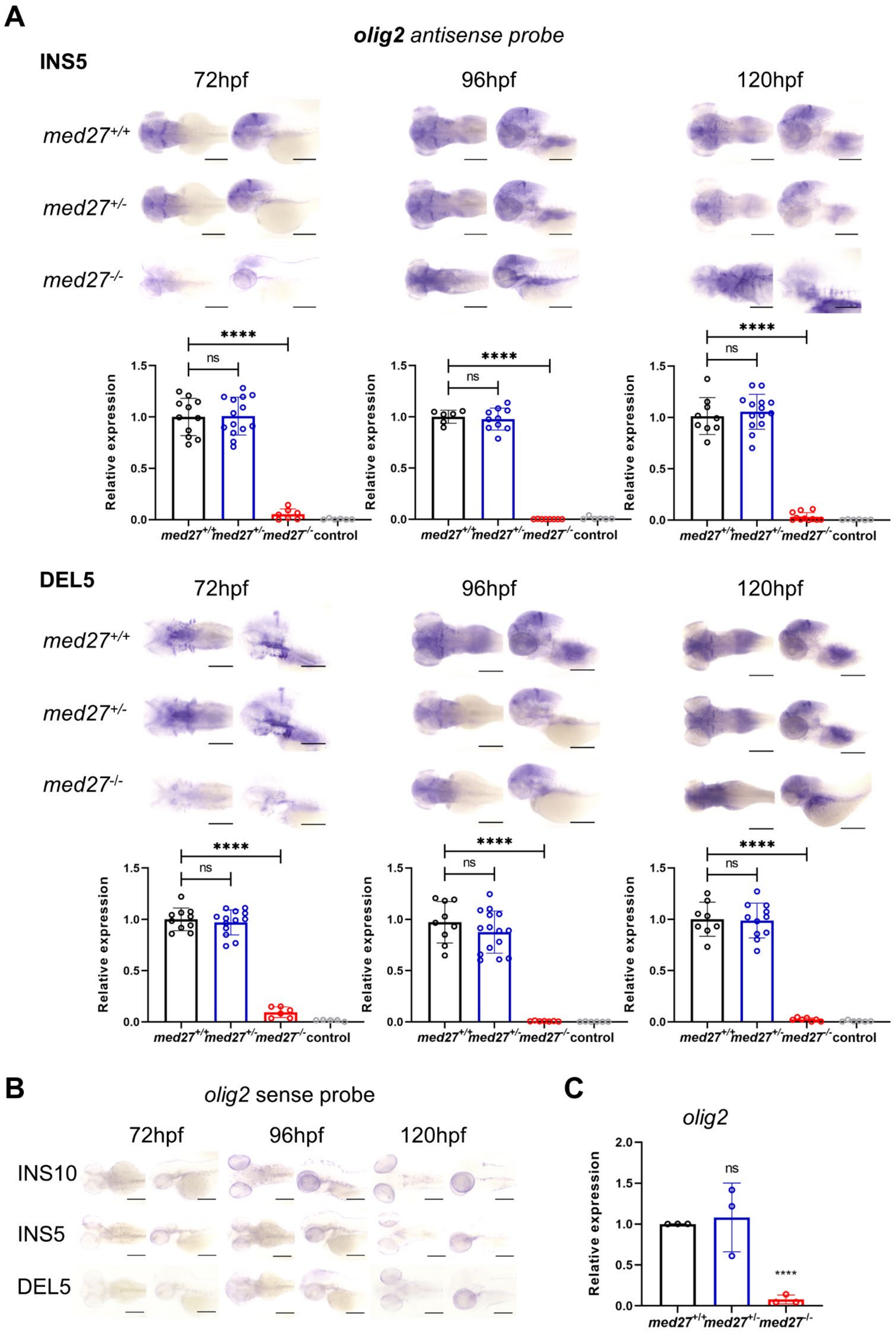
A
olig2 antisense probe
INS5
72hpf
96hpf
120hpf
med27+/+
med27+/-
med27-/-
Relative expression
0.0
0.5
1.0
1.5
ns
****
med27+/+
med27+/-
med27-/-
control
DEL5
B
olig2 sense probe
INS10
INS5
DEL5
C
olig2
2.0

Fig. S6 Related to Fig. 3B. A Representative dorsal and lateral views of WISH for *olig2* at 72 hpf, 96 hpf, and 120 hpf in *med27*$^{+/+}$, *med27*$^{+/-}$, and *med27*$^{-/-}$ larvae from the INS5 and DEL5 mutant lines (INS5 at 72 hpf: *med27*$^{+/+}$ n=11, *med27*$^{+/-}$ n=14, *med27*$^{-/-}$ n=7, control n=6; INS5 at 96 hpf: *med27*$^{+/+}$ n=6, *med27*$^{+/-}$ n=10, *med27*$^{-/-}$ n=8, control n=6; INS5 at 120 hpf: *med27*$^{+/+}$ n=9, *med27*$^{+/-}$ n=14, *med27*$^{-/-}$ n=11, control n=6; DEL5 at 72 hpf: *med27*$^{+/+}$ n=10, *med27*$^{+/-}$ n=13, *med27*$^{-/-}$ n=6, control n=6; DEL5 at 96 hpf: *med27*$^{+/+}$ n=9, *med27*$^{+/-}$ n=15, *med27*$^{-/-}$ n=8, control n=6; DEL5 at 120 hpf: *med27*$^{+/+}$ n=8, *med27*$^{+/-}$ n=11, *med27*$^{-/-}$ n=7, control n=6). **B** WISH images with the *olig2* sense probe as controls. **C** mRNA expression levels of *olig2* in pooled total RNA extracted from 7 dpf larvae from all three mutant lines (INS10, INS5, DEL5). Error bars represent mean ± SD. Statistical analysis was performed using one-way ANOVA. ns: not significant; ****: $P < 0.0001$.

A

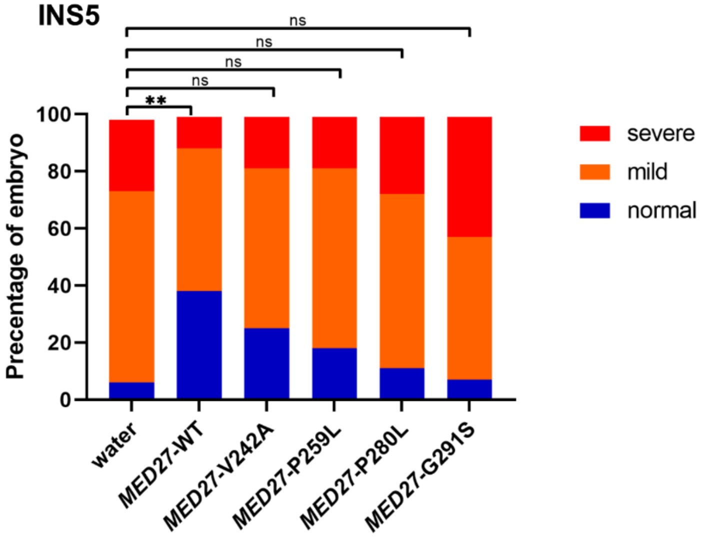

B

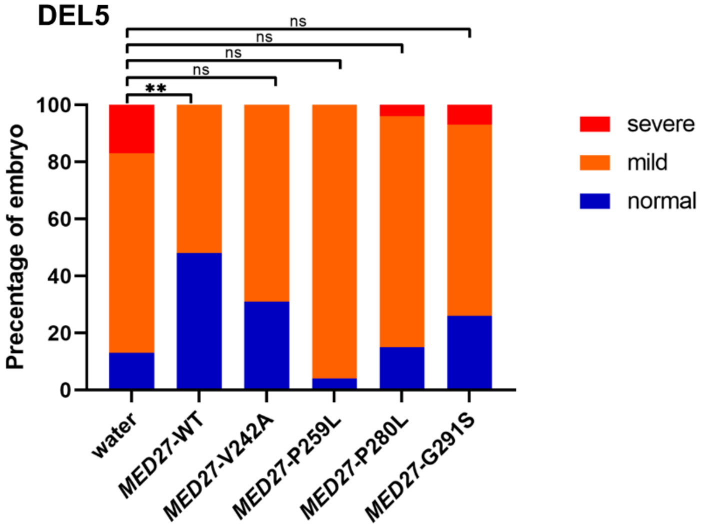

Fig. S7 Related to Fig. 4C. A Distribution of phenotypes after rescue experiments using WT *MED27* mRNA or patient-specific mutant *MED27* mRNA in *med27*$^{-/-}$ fish from the INS5 mutant line. The water-injected group (n=31) served as the control. The following groups received injections of: *MED27*-WT (n=18), *MED27*-V242A (n=16), *MED27*-P259L (n=11), *MED27*-P280L (n=18), and *MED27*-G291S (n=14). **B** Distribution of phenotypes after rescue experiments using WT *MED27* mRNA or patient-specific mutant *MED27* mRNA in *med27*$^{-/-}$ fish from the DEL5 mutant line. The water-injected group (n=24) served as the control. The following groups received injections of: *MED27*-WT (n=27), *MED27*-V242A (n=26), *MED27*-P259L (n=26), *MED27*-P280L (n=27), and *MED27*-G291S (n=27). Phenotype comparisons were analyzed using chi-square tests. ns: not significant; **: P<0.01.
